# Association of Focused Medication Review With Optimization of Psychotropic Drug Prescribing: A Systematic Review and Meta-analysis

**DOI:** 10.1001/jamanetworkopen.2018.3750

**Published:** 2018-10-26

**Authors:** Rory Sheehan, André Strydom, Emma Brown, Louise Marston, Angela Hassiotis

**Affiliations:** 1 Division of Psychiatry, University College London, London, United Kingdom; 2 Forensic and Neurodevelopmental Sciences, Institute of Psychiatry, Psychology and Neuroscience, King’s College London, London, United Kingdom; 3 Department of Primary Care and Population Health, University College London, London, United Kingdom

## Abstract

**Importance:**

Medication review has been proposed to achieve improved use of psychotropic drugs, but benefits have not been confirmed.

**Objective:**

To synthesize evidence for focused psychotropic medication review in medication optimization.

**Data Sources:**

Medline, PsycINFO, EMBASE, and CINAHL Plus were searched from inception to February 2018 using the index terms “drug utilization review” and “psychotropic drugs” and synonyms. Additional articles were retrieved using citation tracking and reference checking.

**Study Selection:**

Full-length, peer-reviewed articles that reported focused psychotropic medication review were included. Inclusion was determined against prespecified criteria and assessed independently.

**Data Extraction and Synthesis:**

Study quality was assessed using National Institutes for Health appraisal tools and informed a structured synthesis of results. Meta-analysis using a random effects model was conducted.

**Main Outcomes and Measures:**

Change in the number or dosage of psychotropic medications, change in clinical parameters, change in patient-reported outcomes, and economic data were collected.

**Results:**

A total of 26 studies met the inclusion criteria. Four studies were randomized clinical trials (n = 712 participants), while the remainder were before-after studies (n = 7844 participants). Most studies were conducted in elderly individuals, people with dementia, and adults with intellectual disability. Focused psychotropic medication review is a complex intervention; the professional(s) involved, target drug, degree of integration with usual care, and participant involvement varied greatly among the studies. Meta-analysis included 3 studies (n = 652 participants). Psychotropic medication review was associated with a reduction in prescribing of psychotropic drugs compared with control (pooled odds ratio, 0.24; 95% CI, 0.14-0.39) in elderly participants with cognitive impairment living in nursing homes. Before-after studies consistently reported a change in psychotropic drug prescribing after medication review, regardless of the population. Studies that reported the effects of psychotropic medication review on clinical outcomes failed to demonstrate benefit. Economic implications of focused psychotropic medication review were not adequately assessed. The quality of evidence is poor and studies are at risk of bias.

**Conclusions and Relevance:**

Focused psychotropic medication review was associated with a reduction in prescribing of psychotropic drugs, but has not been shown to improve clinical outcomes or to provide economic benefit. More robust evidence is needed before programs of focused psychotropic medication review can be recommended as part of routine care for any patient group.

## Key Points

**Question:**

Does focused psychotropic medication review promote medication optimization?

**Findings:**

This systematic review and meta-analysis found that focused psychotropic medication review is associated with reduction in drug prescribing. Advantages for clinical and patient-important outcomes have not been shown.

**Meaning:**

More research using standardized medication review processes and outcome measures is needed before routine programs of focused psychotropic medication review can be recommended.

## Teaser

This systematic review and meta-analysis examines factors associated with focused psychotropic medication review and medication optimization.

## Introduction

The increasing worldwide use of psychotropic drugs and their application beyond licensed indications has attracted close scrutiny.^1,2,3^ At least 1 in 6 adults in the United States is prescribed psychotropic medication,^4^ and high levels of psychotropic drug use are demonstrated in several vulnerable groups, including elderly individuals,^5^ people with dementia,^6^ children and adolescents,^7^ and those with neurodevelopmental disorders, including autism^8^ and intellectual disability.^9,10^ Although undoubtedly of benefit to many individuals, psychotropic drugs are associated with significant adverse drug events that can affect quality of life and result in additional service costs.^11,12^ Spending on psychotropic drugs continues to grow^13,14^ and a high rate of nonadherence leads to significant waste.^15^

Medication optimization is a broad approach aimed at ensuring the safest and most effective use of medications.^16^ The concept has gained traction and has been applied to psychotropic drug prescribing, for example, in the Centers for Medicare & Medicaid Services’ recent efforts to reduce inappropriate antipsychotic prescribing in nursing homes.^17^

Medication optimization encompasses a range of strategies that may be used throughout the medication pathway, including educational interventions, formularies that identify drugs with the greatest perceived overall value, consensus guidelines to direct best practices, benchmarking of prescription rates, and decision aids developed to enable patients to take a more active role in treatment decisions based on their values and preferences. After a drug has been prescribed, optimization includes support for adherence and medication reconciliation. Medication review, a structured and critical evaluation of medication, might have a role in medication optimization, maximizing therapeutic efficacy, mitigating adverse events, and identifying opportunities for decreasing prescribing.^18^ In this systematic review we describe the content and delivery of focused psychotropic medication review and synthesize the evidence for its contribution to medication optimization.

## Methods

A literature search was conducted in Medline, PsycINFO, EMBASE, and CINAHL Plus from inception to February 2018 to identify peer-reviewed original research articles that reported the effect of focused psychotropic medication review on medication optimization outcomes. Search terms included “drug utilization review” (and synonyms) combined with “psychotropic drugs” (and synonyms) (see eTable 1 in the Supplement for an example of full search strategy). Psychotropic drugs were defined in accordance with the World Health Organization Anatomical Therapeutic Chemical Classification System.^19^ Medication review was defined as a structured critical evaluation of medication with an aim to improve safety, efficacy, or cost-effectiveness. Optimization outcomes were intentionally broad. Any study design was included and there were no restrictions on population, setting, language, or date of publication. This study followed the Preferred Reporting Items for Systematic Reviews and Meta-analyses (PRISMA) reporting guideline. The protocol was registered prospectively with PROSPERO (registration No. CRD42017077244).

To limit potential confounding, studies were excluded if they reported a comprehensive medication review (including multiple classes of medication) or conducted a medication review as part of a multimodal intervention where the effect of medication review on reported outcomes could not be distinguished. Short reports, research letters, dissertations, and conference abstracts were not included but prompted a search for full-length articles. Reference lists of included studies and previously published reviews in the field were extensively hand searched to find articles not identified in the database search. The citations of included articles were identified using Google Scholar and considered for relevance.

After exclusion of duplicate records, the titles of all articles were screened by one of us (R.S.), and a randomly selected sample was independently reviewed by another of us (E.B.). They independently reviewed abstracts of remaining studies (and later selected full text) against inclusion and exclusion criteria, with any discrepancies resolved by consensus or discussion with a third member of the research team.

Study quality was rated independently by 2 of us (R.S. and E.B.) using the relevant quality checklist published by the National Institutes of Health^20^ along with a descriptive evaluation of the limitations of each article. Studies received an overall grading of poor, fair, or good quality based on the proportion of applicable checklist items that were met (poor, <30% of items; fair, 30%-60%; good, >60%). Results of the quality appraisal were used to inform a structured evidence synthesis, with higher-quality studies given precedence.

Data were extracted from studies and used to populate summary tables. Type of medication review was classified according to the Task Force on Medicines Partnership and the National Collaborative Medicines Management Services Program (level 1, prescription review; level 2, treatment review; level 3, clinical medication review).^18^ Outcomes were grouped according to theme, allowing comparison between different studies. Measures of psychotropic drug optimization could include changes in medication-related variables, clinical efficacy or adverse drug events, participant-reported outcomes, or economic evaluations. Additional data were sought by contacting authors of included studies, where indicated.

Results were summarized narratively. Numerical data were extracted and, where comparable, means and 95% CIs were calculated around summary statistics. Odds ratios for comparable outcomes reported in randomized clinical trials (RCTs) were entered into a meta-analysis using the *metan* command in Stata, version 14^21^ and using a random-effects model. The *I*^2^ statistic was used to estimate statistical heterogeneity between studies.^22^

## Results

**Figure 1.**
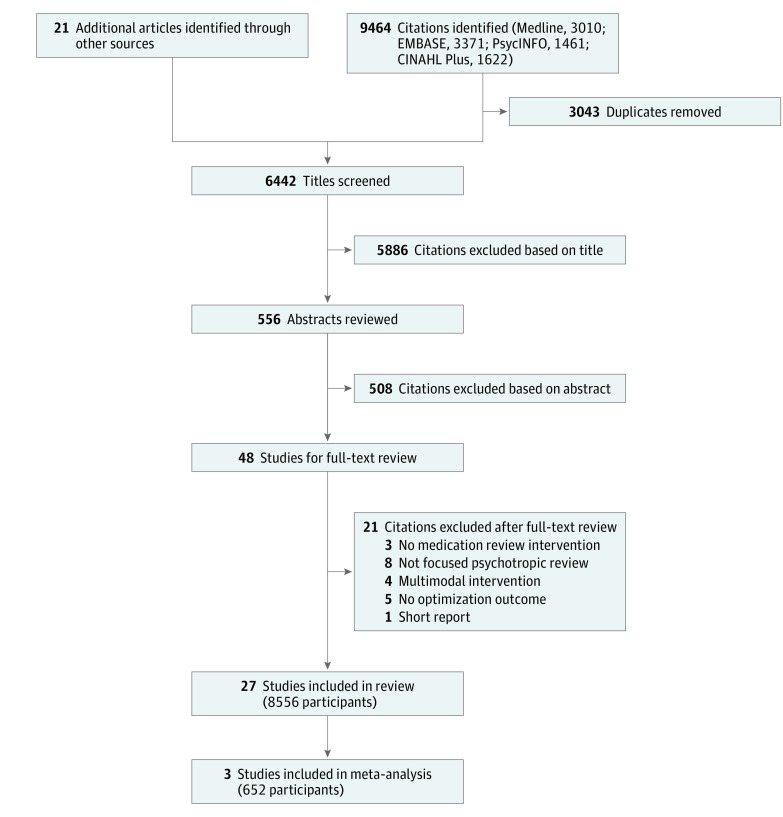
PRISMA Flowchart

The search yielded a total of 9485 articles, of which 27 met inclusion criteria (Figure 1). The results of 1 study were reported in 2 articles.^23,24^ Four studies were cluster RCTs (n = 712 participants)^23,24,25,26,27^ and the remaining 22 were before-after study designs (n = 7844 participants).^28,29,30,31,32,33,34,35,36,37,38,39,40,41,42,43,44,45,46,47,48,49^ Studies were conducted in North America (15 studies),^28,30,31,32,33,35,36,37,39,42,43,46,47,48,49^ Europe (10 studies)^23,25,26,27,29,38,40,41,44,45^ and Australasia (1 study).^34^ A total of 19 studies^23,26,27,28,29,30,31,32,33,34,36,37,39,40,41,43,46,47,48^ were conducted in institutional settings and reported psychotropic medication review of people with intellectual disability (9 studies; 1054 participants)^28,29,30,31,32,33,34,35,36^ or those with dementia (6 studies; 3664 participants).^23,37,38,39,40,41^ Meta-analysis included 3 studies^28,30,34^ (n = 652 participants).

Details of included studies are in eTable 2 in the Supplement. When assessed against objective criteria, most research was at medium to high risk of bias. Key methodological problems encountered across studies were single group design, reporting bias, lack of measures of implementation fidelity, lack of objective and validated outcome measures, short follow-up times, and limited (or absent) statistical analysis. Several studies made claims that were not supported by the findings.

### Content and Delivery of Psychotropic Medication Review

The focused psychotropic medication reviews fell into 3 major categories. The first was one-off medication review, usually undertaken by a single professional and including a single class of psychotropic drug.^23,25,38,40,41,42,43,44,45^ The second model was a longitudinal program of regular medication review, often by a multidisciplinary team who reviewed the participant’s psychotropic drug regimen in a series of meetings.^26,27,28,29,30,31,32,33,34,35,36,37,39^ The third type of focused psychotropic medication review was 2-stage, in which those at high risk of suboptimal drug therapy were identified using a rule applied to the electronic patient record and then directed to clinician medication review.^46,47,48,49^ Within this categorization, the configuration of focused psychotropic medication review varied considerably from remote review by a third party to detailed medication review with access to the patient’s full clinical notes and the patient’s direct input to the process (Table 1).^18,23,24,25,26,27,28,29,30,31,32,33,34,35,36,37,38,39,40,41,42,43,44,45,46,47,48,49^ Medication reviews were most often organized according to local protocols, but specific conduct of the medication review was commonly not reported.

**Table 1.** Focused Psychotropic Medication Review—Summary of Content and Delivery

Source	Participants, Setting	Psychotropic Drugs Reviewed	Professionals Involved	Patient or Patient Representative Involvement	Review Delivery	Guidelines and Instruments Used	Review Level^18^
**One-Off Medication Reviews**							
Ballard et al,^23^ 2016, and Ballard et al,^24^ 2017	277 People with dementia, nursing home	Antipsychotic drugs	Physician	NR	General practitioner or psychiatrist performed antipsychotic review using clinical guidelines to determine appropriateness and direct withdrawal attempts	NICE dementia guidelines; Alzheimer Society guidelines	CD
Moncrieff et al,^25^ 2016	60 People with severe mental illness, community	Antipsychotic drugs	Physician and care coordinator (nurse, social worker, occupational therapist)	Yes: patient	Patients used a medication review tool with their care coordinator prior to a psychiatrist appointment; the tool incorporated perceived benefits and disadvantages of antipsychotic drugs and desired changes, which could be discussed with the prescriber	Medication Review Tool (developed for the study)	3
Gallimore et al,^42^ 2016	144 Children and adults with mental illness, community	Psychotropic drugs	Pharmacist	No	Pharmacist reviewed medication record and electronic health record 1-3 mo after psychiatrist appointment; drug monitoring reviewed against best practice guidelines and potential for drug-drug interactions assessed using drug interaction database; recommendations sent to prescriber	American Psychiatric Association Practice Guidelines, Mount Sinai Conference Consensus recommendations, Development Conference on Antipsychotic Drugs and Obesity and Diabetes guidelines used to define monitoring standards	1
Prentice and Wright,^41^ 2014	3165 Older adults, nursing home	Antipsychotic drugs	Pharmacist, care staff, physician	No	Pharmacist reviewed symptoms, adverse effects, and medication-related information, discussed with care staff, and made recommendations to physician	NICE guidelines, standard data collection form to inform decision making	2
Gemelli et al,^43^ 2016	34 Older adults, nursing home	Sedative and hypnotic drugs	Pharmacist	No	Pharmacist reviewed medication records and, where indicated, made recommendations (dose reduction, drug discontinuation, reevaluation of symptoms, or switch to alternative drugs) to the prescriber	No formal guidelines or standard instruments used in this medication review	1
Child et al,^38^ 2012	70) People with dementia, care home or community	Antipsychotic drugs	Pharmacist	Yes: patient and family	Pharmacist reviewed medication record and clinical records and discussed changes to antipsychotic prescribing with general practitioner, care staff, and patient (±family)	No formal guidelines or standard instruments used in this medication review	3
Johnson et al,^44^ 2012	2849 Adults, community	Antidepressant drugs	Physician	Yes: patient	Physician completed face-to-face medication review	No formal guidelines or standard instruments used in this medication review	3
Napolitano et al,^45^ 2012	32 Adults, community	Antidepressant drugs	Nurse	Yes: patient	Nurse prescriber completed face-to-face medication review including illness- and medication-related variables, patient understanding and beliefs, and risk assessment	Patient Health Questionnaire, Generalized Anxiety Disorder Scale, Work and Social Adjustment Scale, Phobic Scale	3
**Regular Medication Review Programs**							
Jordan et al,^26^ 2015	41 Elderly, nursing home	Psychotropic drugs	Nurse	Yes: patient and family	Monthly nurse review according to a checklist incorporating psychotropic drug adverse effects other and unmet needs; completed with patient and acting as a prompt to further activity, including prescriber medication review	West Wales Adverse Drug Reaction profile	3
Patterson et al,^27^ 2010	334 Elderly individuals, nursing home	Psychotropic drugs	Pharmacist, physician	Yes: patient and family	Monthly pharmacists reviewed patient records and interviewed patients and family to identify drug-related problems and used an algorithm to identify potentially inappropriate psychotropic drug prescribing; pharmacist recommendations discussed with physician and drug decisions made	Fleetwood algorithm for appropriateness of psychotropic drug prescription	3
Bach et al,^37^ 2017	20 People with dementia, nursing home	Antipsychotic drugs	Pharmacist	No	Monthly pharmacist screened medication charts against criteria for appropriate antipsychotic use and made recommendations to the physician	Antipsychotic Use Survey Tool was used to determine appropriate and inappropriate antipsychotic prescribing	1
Morrison,^40^ 2009	22 Older adults, nursing home	Antipsychotic drugs	General practitioner	NR^a^	General practitioner completed structured review of antipsychotic prescribing supported by a checklist based on NICE guidance every 6 mo	No formal guidelines or standard instruments used in this medication review	CD
Dahl et al,^39^ 2008	110 People with dementia, long-term care	Psychotropic drugs	Nurse, social worker, pharmacist, physician	Yes: family	Multidisciplinary team gather information using a standardized psychotropic assessment form covering symptoms, behavior, adverse effects, and patient and family concerns every 6 mo; followed by a multidisciplinary team meeting where recommendations to optimize prescribing are agreed and sent to the prescriber	Psychotropic Assessment Tool	3
Branford,^29^ 1996	198 People with intellectual disability, institution	Antipsychotic drugs	Nurse, psychiatrist, pharmacist	No	Regular multidisciplinary meeting to review diagnosis, behavior, and medication prescribing; prescribing decisions made by consensus	Aberrant Behavior Checklist, Psychopathology Instrument for Mentally Retarded Adults, Reiss screen	2
Bisconer et al,^28^ 1995	80 People with intellectual disability, institution	Psychotropic drugs	Physician, pharmacist, psychologist, nurse, other professional staff, lay participants	No	Multidisciplinary meetings to discuss presentation, drug adverse effects, and broader treatment plan every 6 mo; changes to prescribing made by consensus	Standard report (no validated instruments)	2
Jauernig et al,^34^ 1995	25 People with intellectual disability, institution	Psychotropic drugs	Pharmacist, physician, psychologist, care staff, clinical manager	No	Multidisciplinary meetings every 2 mo to discuss presentation and progress, review data collected on standardized forms, and agree drug recommendations to be made to treating physician	Behavior monitoring record forms, Aberrant Behavior Checklist, adverse effect monitoring checklist	2
Glaser et al,^32^ 1986	28 People with intellectual disability, institution	Antipsychotic drugs	Physician, nurse, pharmacist, psychologist, care staff, administrator	No	Monthly multidisciplinary team review including indication for medication, symptoms, alternative treatments, and medication response; recommendations made	No formal guidelines or standard instruments used in this medication review	2
Marcoux,^36^ 1985	255 People with intellectual disability, institution	Psychotropic drugs	Physician, psychologist, nurse, pharmacist	No	Multidisciplinary meetings every 3 mo to review symptoms, adverse effects, and other information and inform medication decisions	Standard data sheets completed	2
Lepler et al,^35^ 1993	12 People with intellectual disability, community	Psychotropic drugs	Nurse, psychologist, care staff, physician	Yes: family or advocate	Multidisciplinary review every 3 mo of clinical presentation, medication response, and adverse effects, laboratory monitoring, alternative interventions, and other factors leading to drug recommendations based on team consensus; final decisions are a combination of team recommendations, patient and family preference, and physician opinion	No formal guidelines or standard instruments used in this medication review	3
Ferguson et al,^31^ 1982	97 People with intellectual disability, institution	Antipsychotic drugs	Physician, psychologist, social worker, nurse, pharmacist, care staff	No	Monthly multidisciplinary review of target symptoms and medication adverse effects with data (counts of challenging behavior) used to direct drug dose changes according to a specified protocol	No formal guidelines or standard instruments used in this medication review	2
Inoue,^33^ 1982	251 People with intellectual disability, institution	Psychotropic drugs	Pharmacist, physician, nurse, care staff	No	Monthly pharmacist collected data on patient condition, response to treatment, drug adverse effects presented at multidisciplinary meetings; pharmacist recommendations for treatment discussed and accepted or declined	Standard data forms used to inform reviews	2
Ellenor et al,^30^ 1977	208 People with intellectual disability, institution	Psychotropic drugs	Physician, pharmacist, nurse, psychologist, sociologist, therapist	No	Pharmacist collected data every 3 mo on drug history, interactions, adverse effects, clinical presentation, response to treatment, and made recommendations that were discussed and accepted or declined at multidisciplinary meetings	Data collected on a standard form	2
**Electronic Identification of Prescribing Followed by Clinician Medication Review**							
Donat,^46^ 2006	People with mental illness, hospital^a^	Psychotropic drugs (as-needed use)	Psychiatrist, psychologist	No	Automated identification of patients receiving as-needed medication ≥3 times a week followed by case review by psychiatrist and psychologist using a semistructured form to guide decisions; further review by a senior management committee in some cases	Local guidelines	2
Seltzer et al,^49^ 2000	Adults and children, community^a^	Sedatives and hypnotics	Physician	NR^a^	Automated identification of patients prescribed long-term or high-dose sedatives or intraclass polypharmacy followed by letter to prescriber to prompt review of medication (this stage of medication review not well described)	No formal guidelines or standard instruments used in this medication review	1
Craig et al,^47^ 1984	People with mental illness, hospital^a^	Psychotropic drugs	Physician	No	Automated identification of patients receiving high or low drug doses or polypharmacy followed by clinical review by 2 physicians to judge appropriateness of prescribing; further review by senior physicians when agreement not reached	No formal guidelines or standard instruments used in this medication review	2
Laska et al,^48^ 1980	People with mental illness, hospital^a^	Psychotropic drugs	Physicians	No	Automated identification of patients receiving high or low drug doses or polypharmacy followed by drug review by 2 physicians and consultation with a peer group, if necessary	No formal guidelines or standard instruments used in this medication review	2

Abbreviations: CD, cannot determine; NICE, National Institute for Health and Care Excellence; NR, not reported.

^a^ No. of participants not given.

Studies reported review of antipsychotic drugs (9 studies),^23,25,29,31,32,37,38,40,41^ sedatives and hypnotics (2 studies),^43,49^ antidepressants (2 studies),^44,45^ or several psychotropic drug classes concomitantly (13 studies).^26,27,28,30,33,34,35,36,39,42,46,47,48^ A range of medical and other health professionals were involved in medication reviews, with the most consistent representation being by clinical pharmacists. A minority of reviews incorporated involvement of the patient or family or advocate.

Several studies reported standardized methods of data collection that were used to inform medication review, although few used validated instruments to measure clinical variables. Most focused psychotropic medication reviews relied on implicit decision making and clinician judgment, rather than explicit measures of drug appropriateness and best practice guidelines.

### Medication-Related Outcomes of Psychotropic Medication Review

**Figure 2.**
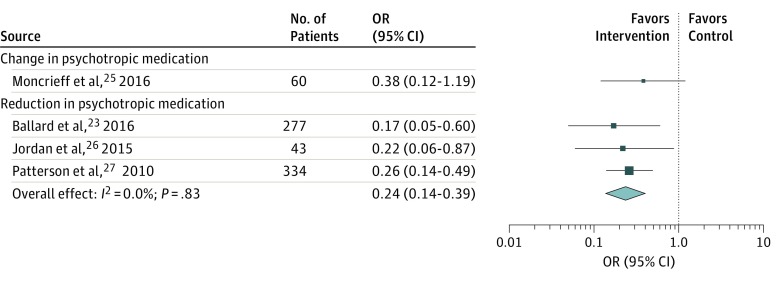
Forest Plot Showing Odds of Change in Antipsychotic Medication and Odds of Reduction in Psychotropic Medication Following Focused Medication Review vs Treatment as Usual The size of the data markers is determined by weight from random-effects analysis. OR indicates odds ratio.

A measure of change in psychotropic drug prescribing after focused psychotropic medication review was the most consistently reported medication-related outcome. Ballard et al,^23^ Jordan et al,^26^ and Patterson et al^27^ report a significant effect of focused psychotropic medication review in reducing psychotropic drug prescribing in cognitively impaired elderly residents of nursing homes (pooled odds ratio, 0.24; 95% CI, 0.14-0.39) (Figure 2).^23,25,26,27^ Additional data obtained from Moncrieff et al^25^ showed a nonsignificant tendency to greater change in antipsychotic medication among adults with severe mental illness undergoing outpatient focused psychotropic medication review conducted by their usual psychiatrist than among those receiving standard care (odds ratio, 0.38; 95% CI, 0.12-1.19).

**Figure 3.**
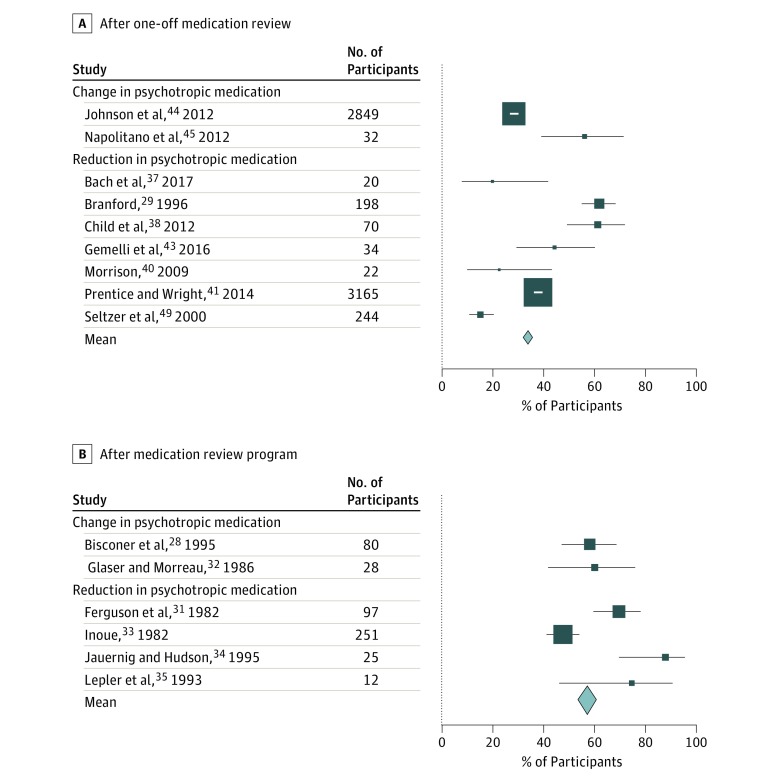
Proportion of Participants of Included Studies With Change in Psychotropic Medication Prescription A, After one-off medication review. B, After medication review program. The size of the data markers is based on the number of participants, and the error bars indicate 95% CIs.

One-off focused psychotropic medication review was associated with mean of 34.0% (95% CI, 32.9%-35.2%) of participants having a change in medication prescription (Figure 3A).^29,37,38,40,41,43,44,45,49^ Four before-after studies report the effect of focused medication review on antipsychotic drugs prescribed for behavioral and psychological symptoms of dementia.^37,38,40,41^ These reviews, conducted by either a pharmacist^37,38,41^ or general practitioner,^40^ were associated with a reduction or discontinuation of antipsychotic drugs in 20% to 61% of participants.

Gemelli et al^43^ investigated pharmacist review of sedative and hypnotic medication in elderly people living in a nursing home. The intervention was associated with dose reduction or discontinuation in 49% of the sample by follow-up at 3 months.

Two before-after studies addressed the effect on drug prescribing of clinical review of long-term antidepressant drug therapy in community-dwelling adults. One large Scottish study (N = 2849) found that 28.5% of individuals taking antidepressants had a medication change after in-person review by their general practitioner, with most changes being drug discontinuation or dose reduction.^44^ A similar study, conducted on a much smaller scale (N = 32), reported that medication change followed just above half of the medication reviews conducted by a specialist nurse prescriber.^45^

The 4 studies that used electronic prescribing records to identify prescribing that fell outside defined guidelines to generate alerts, prompting clinician-focused psychotropic medication review, all report that the process was associated with improved rates of guideline-compliant prescribing.^46,47,48,49^

Several studies report the association of a program of multidisciplinary medication review with psychotropic prescribing. In all but 1 case, these studies were conducted before 2000 and focused on the use of psychotropic drugs for challenging behavior in adults with intellectual disability, most of whom were receiving long-term institutional care.^28,29,30,31,32,33,34,35,36^ The quality of these studies is poor to fair, yet together they report results of psychotropic medication review of a relatively homogeneous group of more than 1000 adults (most with severe to profound intellectual disability and behavior disturbance living in large institutional facilities), with follow-up of between 6 months and 4 years. Figure 3B^28,31,32,33,34,35^ shows the proportion of participants in these studies undergoing a reduction or change in psychotropic medication after the review programs, where this metric is given or can be extrapolated from the published results (mean proportion changing prescription, 57.6%; 95% CI, 53.2%-62.0%). These studies demonstrate the association of focused psychotropic medication review with medication change in a potentially overmedicated group; most changes were dose reductions or discontinuations.

Studies that do not report the proportion of participants with medication change still report an association with either reduction of overall psychotropic prescribing at the group level^30,36^ or reduced rates of polypharmacy.^28,34^ Ellenor and Frisk^30^ demonstrate a reduction of 37% in psychotropic prescription items during the course of a 2-year regular medication review and Marcoux^36^ found that their process of psychotropic medication review was associated with a mean dose reduction of 17% in individuals receiving antipsychotic drugs. An exception to these findings is a more recent study by Dahl et al,^39^ who report the results of a thorough multidisciplinary psychotropic review in people with dementia who lived in nursing homes. The review was associated with minimal change in prescribing of any class of psychotropic drug, although interpretation is limited by movement of participants into and out of the intervention group.

Psychotropic polypharmacy among participants before and after the medication review program was reported by 3 studies.^28,30,34^ All 3 studies report a substantial reduction in the total volume of psychotropics associated with focused psychotropic medication review.

### Clinical and Patient-Related Outcomes of Psychotropic Medication Review

Several disparate clinical outcomes were measured in twelve studies (Table 2).^23,24,25,26,27,28,29,30,32,33,34,42^ Ballard et al^23^ reported significantly more neuropsychiatric symptoms at 9-month follow-up in people with dementia receiving antipsychotic review compared with controls (group difference in Neuropsychiatric Inventory score, 7.37; 95% CI, 1.53-13.22). Furthermore, people receiving the intervention demonstrated a nominal worsening in health-related quality of life (measured with the DEMQOL: Dementia Quality of Life measure), which did not reach statistical significance (group difference, 4.54; 95% CI, –9.26 to 0.19).^24^ There was no difference in agitation, depression, or mortality between groups.

**Table 2.** Clinical Outcomes of Focused Psychotropic Medication Review

Source	Clinical Outcome	Measure	Result
**Randomized Clinical Trials**			
Ballard et al,^23^ 2016	Neuropsychiatric symptoms	Neuropsychiatric Inventory	Difference between intervention and control groups favors control 7.37 (95% CI, 1.53-13.22; *P* = .02)^a^
	Agitation	Cohen-Mansfield Agitation Inventory	Difference between intervention and control groups: 4.60 (95% CI, −1.43 to 10.63; *P* = .13)
	Depression	Cornell Scale for Depression in Dementia	Difference between intervention and control groups: −1.70 (95% CI, −4.29 to 0.90; *P* = .19)
	Mortality	Death	OR, 0.67 (95% CI, 0.39-1.14; *P* = .15)
Ballard et al,^24^ 2017	Proxy health-related quality of life	DEMQOL: Dementia-Related Quality of Life measure	Difference between intervention and control groups: 4.54 (95% CI, −9.26 to 0.19; *P* = .06)
Moncrieff et al,^25^ 2016	Clinical symptoms of severe mental illness	Brief Positive and Negative Syndrome Scale	Difference between intervention and control groups: 0.13 (95% CI, −2.20 to 2.48)
	Antipsychotic drug adverse effects	Liverpool University Neuroleptic Side-Effect Rating Scale	Difference between intervention and control groups: −0.42 (95% CI, −8.12 to 7.29)
	Medication adherence	Medication Adherence Questionnaire	Difference between intervention and control groups favors intervention: −0.44 (95% CI, −0.76 to −0.11)^a^
	Confidence in participating in clinical discussions and decisions	Decision Self-Efficacy Scale	Mean difference between intervention and control group: −4.16 (95% CI, −9.81 to 1.49)
	Patient satisfaction	Client Satisfaction Questionnaire	Difference between intervention and control groups: −0.29 (95% CI, −3.04 to 2.45)
	Attitude toward medication	Drug Attitude Inventory	Difference between intervention and control groups: 1.65 (95% CI, −0.09 to 3.40)
Jordan et al,^26^ 2015	Drug-related problems addressed (adverse effects)	Counts	Difference between intervention and control groups favors intervention: 3.34 (95% CI, 2.57-4.11; *P* < .001)^a^
	Functional ability	Bristol Activities of Daily Living Scale	Mean difference between intervention and control group: 0.45 (95% CI, −0.47 to 0.93; *P* = .52)
	Dementia psychopathology	Manchester and Oxford Universities Scale for the Psychopathological Assessment of Dementia	Difference between intervention and control groups: 4.67 (95% CI, −0.04 to 2.78; *P* = .06)
Patterson et al,^27^ 2010	Falls	Rate	16.3 falls/100 person-mo in intervention group vs 11.4 falls/100 person-months in control group (*P* = .09)
**Before-After Study Designs**			
Gallimore et al,^42^ 2016	Up-to-date laboratory monitoring	Proportion of participants	54.1% (before); 72.1% (after) (*P* < .001)^a^
	At risk of drug-drug interaction	Proportion of participants	43.8% (before); 24.3% (after) (*P* < .001)^a^
	Movement adverse effect monitoring	Proportion of participants	75.0% (before); 63.5% (after) (*P* = .21)
Branford,^29^ 1996	Clinical presentation	Subjective assessment	25% undergoing medication change had “good” outcome, 43% “poor” outcome, 32% “unclear” outcome
Jauernig et al,^34^ 1995	Challenging behavior	Frequency counts	Mean daily frequency of challenging behavior lower after the intervention than at baseline in 80%
Bisconer et al,^28^ 1995	Challenging behavior	Frequency counts	Mean decrease in challenging behavior after intervention
	Reported medication adverse effects	Proportion of participants	(n = 11 [14%]) before intervention, (n = 8 [10%]) after intervention
Glaser et al,^32^ 1986	Aggressive challenging behavior	Frequency counts	No significant difference between intervention and control groups^b^
Inoue,^33^ 1982	Clinical presentation	Subjective assessment	“Positive change” in 96.5% receiving intervention, “negative” change in 3.5%
Ellenor et al,^30^ 1977	Challenging behavior	Aberrant Behavior Checklist	“Slight increase” in challenging behavior but no significant difference between intervention and control group^b^

Abbreviation: OR, odds ratio.

^a^ Results statistically significant at *P* < .05.

^b^ These studies had control groups only for the secondary outcome of change in challenging behavior.

The medication review intervention tested by Moncrieff et al^25^ was co-designed with people with severe mental illness with the aim of increasing patient involvement and agency in decision making regarding antipsychotics. There was no difference in scores on the Decision Self-Efficacy Scale between those randomized to receive the intervention and those receiving treatment as usual, suggesting that the focused psychotropic medication review did not improve patients’ confidence in discussions or decisions about psychotropic medication. Those in the review group demonstrated a tendency to greater medication adherence but no significant difference was found in other secondary outcome measures of patient satisfaction, attitude toward psychotropic drugs, symptoms of psychosis, or adverse effects of antipsychotic drugs between groups at follow-up 2 to 4 weeks after the review meeting. However, fidelity of implementation was poor and the study tested only feasibility and therefore was not powered to detect effect sizes.

Jordan et al^26^ reported that more medication-related problems were identified and addressed with nurse-led medication review than without. There was no significant difference in change in psychopathologic characteristics of dementia, behavior changes, or functional ability between groups during the study period. Longer-term outcomes on patient health or well-being were not assessed.

Patterson et al^27^ measured the rate of falls in a group of individuals with dementia who needed a high level of care. The reductions in inappropriate psychotropic drug use in the intervention group vs the control group did not translate to a difference in the rate of falls between groups (11.4 falls per 100 person-months in the control group vs 16.3 falls per 100 person-months in intervention group; *P* = .09), although the method of recording falls was subject to inaccuracies and the authors note that the study was underpowered.

Gallimore et al^42^ reviewed the potential for a focused psychotropic medication review by a remote pharmacist, conducted several weeks after out-patient psychiatry consultation, to improve rates of routine adverse drug event monitoring. The focused psychotropic medication review was associated with an increase in the proportion of participants with up-to-date laboratory monitoring and significantly reduced the proportion of those at risk of drug-drug interactions but had no effect on the proportion of participants who were monitored for movement adverse effects. The actual benefit in terms of adverse drug event rates was not measured.

The study by Bisconer et al^28^ was the only one, to our knowledge, to report rates of adverse drug events, albeit with a basic and unvalidated method. The proportion of the cohort with any physician-observed adverse effect decreased from 14% at baseline to 10% after the review program, but the small number of participants is a major limitation of this study.

Four studies report change in challenging behavior as a result of antipsychotic review and reduction programs in institutions for people with intellectual disability.^28,30,32,34^ These studies report a decrease or no change in challenging behavior associated with the delivery of the program. The authors concluded that many psychotropic drugs given in this population can be stopped without causing further deterioration in behavior.

### Economic Outcomes of Psychotropic Medication Review

Five studies^26,30,36,44,45^ reported descriptive cost data in terms of savings made after focused psychotropic medication review. None of the 5 studies conducted comprehensive economic evaluation (eTable 3 in the Supplement).

## Discussion

Psychotropic medication plays a central role in the treatment of mental disorders, yet remains the subject of debate. Treatment benefits must be balanced against adverse drug events, which are both common and distressing.^50^ Rising rates of prescription of psychotropic drugs are observed worldwide, despite the modest effect size of these medications for most indications^51^ and the increasing availability of and evidence for nonpharmacologic interventions. From an economic perspective, the estimated US annual expenditure on psychotropic drugs of $30 billion^52^ must be viewed in the context of nonadherence rates of up to 65%,^53^ which contributes to significant waste of health care resources. On a personal level, patient views and preferences should be respected, but many report feeling disempowered and excluded from decisions about psychotropic medication.^54^ Medication optimization aims to address these tensions through a variety of strategies, including medication review.

Our review of the literature shows that programs of focused psychotropic medication review have attracted ongoing interest and have been instituted across different settings during the past 4 decades. There is considerable diversity in how focused psychotropic medication review has been delivered.

Meta-analysis of 3 RCTs demonstrated a significantly greater likelihood of psychotropic drug reduction with focused psychotropic medication review than with treatment as usual in elderly individuals with dementia living in nursing homes.^23,26,27^ Results from several uncontrolled before-after studies seem to support this finding by reporting an association between medication review and change in drug prescribing, irrespective of participant group. This finding indicates potential for improved prescribing after medication review and, although it seems logical that a change in medication after a critical evaluation will be beneficial, the clinical gains after focused psychotropic medication review cannot be assumed. Many studies did not measure benefits (or harms) associated with medication review. A change in drug prescribing is an intermediary outcome that offers only a crude measure of medication optimization; only 1 study objectively assessed medication appropriateness against consensus guidelines.^27^ One RCT reported identification of a greater number of adverse drug events as a result of focused psychotropic medication review, but not the resolution of these adverse events.^26^ Two RCTs failed to demonstrate any benefit of medication review on clinically meaningful parameters^25,27^ and 1 RCT reported deterioration in patients’ neuropsychiatric symptoms after medication review and subsequent reduction in drug prescribing.^23,24^

The medication optimization approach is intrinsically patient-centric^55^ and medication review can provide an opportunity to explore patient beliefs and preferences and to reach shared treatment decisions. However, with 1 notable exception,^25^ our review highlights a major lack of patient input and personalization in existing focused psychotropic medication reviews, which represents a significant barrier to achieving true medication optimization. This finding may reflect the age of several of the studies and a prevailing paternalism in medication decision making in those prescribed psychotropic drugs.^56^

The potential for financial savings is a strong motivator for medication optimization, yet investigation of the economic implications and resource use of psychotropic medication review has largely been neglected. Any cost savings from reductions in prescribing and avoidance of adverse drug events must be offset against the initial outlay of performing the medication review, additional activity generated (eg, referrals to another health care professional or increased monitoring mandated by drug changes), and switches to more expensive drugs or preparations. In these terms, our findings support calls for more attention to be paid to economic evaluation of medication review.^57,58^

Most of the studies included in our review were conducted in elderly individuals, people with dementia, or adults with intellectual disability, in many cases residing in institutional facilities. People in these groups are at high risk of suboptimal psychotropic use and overuse and may lack the capacity to consent to treatment decisions, yet their underrepresentation in controlled clinical trials leads to a lack of empirical evidence and data-driven prescribing guidelines. Individual regular and pragmatic psychotropic medication review may have the most to offer to these groups, where advantages of medications are less well established and adverse effects are common and idiosyncratic. However, to realize this potential, the quality and reproducibility of future studies must be improved. Although recent studies are more robust methodologically, the overall risk of bias is high, with limitations conferred by both study design and reporting. Future trials should ensure standardized reporting of the intervention (eg, using the TIDieR [Template for Intervention Description and Replication] checklist^59^) and address the feasibility and acceptability of medication review interventions, as their implementation in routine care may be complex^25^ and they may not always be welcomed by patients or their caregivers.^60^ Moreover, inconsistencies in terminology should be resolved and the concept of medication review clearly delineated from other attempts to optimize psychotropic prescribing.^61,62^

Data in the included studies were not sufficiently detailed to enable subgroup analysis of medication reviews, beyond grouping interventions by one-off medication review and ongoing review programs. Multidisciplinary review programs, conducted mostly with participants with intellectual disability living in institutional care, tended to be associated with the greatest proportion of participants changing medication, although this finding could be a function of the high level of psychotropic use in this group.^63^

There is no universally accepted standard procedure for medication review.^16,64,65^ Although best practice advice and consolidated tools have been developed to guide medication review^66,67^ and define potentially inappropriate prescribing,^68,69,70^ few of the medication reviews were informed by a theoretical model. There is the potential for electronic mechanisms, including e-prescribing and the electronic patient record, to support medication review, in a way which does not yet seem to have been investigated fully.^71^

Our findings do not suggest a professional discipline that should lead psychotropic medication review, although third-party reviews by a professional (usually a pharmacist) external to the usual team might be difficult to embed in routine care. As few as one-third of medication recommendations made by pharmacists are actioned by prescribers,^37^ and nonprescribers conducting reviews often report lacking influence,^72^ highlighting the importance of good interprofessional communication in complex medication review interventions.

Existing studies and reviews that evaluate the effect of medication review (in other populations and focused on other drug types) similarly conclude that medication review can influence proximal medication-related outcomes but demonstrate weak evidence of benefit in clinical and patient outcomes.^66,67,73,74^ Despite this finding, medication review is recommended by guidelines as part of routine care for several groups at high risk of suboptimal prescribing and adverse drug events, including elderly individuals, those with chronic conditions, and those receiving polypharmacy.^16,75^

This systematic review and meta-analysis was of focused psychotropic medication review. As an isolated intervention, focused medication review has limitations. Comprehensive medication review, in which all medications are reviewed simultaneously regardless of indication, can highlight drug-drug interactions that might be missed by focusing on psychotropics alone. In practice, however, psychotropic drug prescribing is often performed by specialists who may not feel equipped to review medication for physical health conditions. A treatment review that also incorporates response to nonpharmacologic interventions is attractive, but might be difficult to protocolize where treatment aims span different dimensions.

### Strengths and Limitations

To our knowledge, this is the first systematic review to synthesize the evidence for focused psychotropic medication review. We applied few limits to the search and included varied study designs. We identified several additional articles through hand searches; this approach is likely to reflect the nebulous nature of the intervention and a lack of consistent terminology, as well as poor indexing of older studies. Members of the research team independently appraised studies against prespecified inclusion criteria and judged study quality using published frameworks. We conducted meta-analysis and calculated pooled effects where possible.

The deficits of the primary literature limit the strength of our conclusions about the benefits of psychotropic medication review. Most studies included were uncontrolled and prone to bias and confounding, and it is difficult to attribute causality in before-after designs. It is impossible to blind participants and practitioners to the intervention, although blinding might not be essential where outcomes (eg, prescribing rates) are objective. Clinical outcomes of medication review may only become apparent after some time; thus, the limited follow-up in the included studies is a major problem. Diversity in outcome measures and reporting precluded more extensive meta-analysis. There is potential for publication bias to skew the results of this review and there were insufficient data to assess this risk statistically. We did not search the gray literature.

## Conclusions

Focused psychotropic medication review as a structured and critical evaluation of a patient’s drug therapy has the potential to contribute to medication optimization. Despite much attention and incorporation into routine care, the evidence for focused psychotropic medication review as a stand-alone intervention is weak and it has not been shown that changes in psychotropic prescribing associated with focused psychotropic medication review translate to improved clinical and patient-important outcomes. High-quality research is essential before a routine program of focused psychotropic medication review can be recommended, either in general or special populations. Standardization of nomenclature, processes, and an agreed common set of outcomes that prioritizes patient-important measures is needed. This might be achieved with the creation of a national or international collaborative medication review research network.

## Notes

Supplement. **eTable 1.** Example Search Strategy (PsycINFO, American Psychological Association) **eTable 2.** Summary of Included Studies **eTable 3.** Economic Measures and Outcomes of Medication Review **eReferences**
