# Supplementary material for: Association of Focused Medication Review With Optimization of Psychotropic Drug Prescribing: A Systematic Review and Meta-analysis
Source: JAMA Netw Open. 2018 Oct 26;1(6):e183750. doi: 10.1001/jamanetworkopen.2018.3750 (PMC6324597; doi:10.1001/jamanetworkopen.2018.3750)
Supplement: Supplement. — eTable 1. Example Search Strategy (PsycINFO, American Psychological Association) eTable 2. Summary of Included Studies eTable 3. Economic Measures and Outcomes of Medication Review eReferences [file jamanetwopen-1-e183750-s001.pdf]

Supplementary Online Content

Sheehan R, Strydom A, Brown E, Marston L, Hassiotis A. Association of focused medication review with optimization of psychotropic drug prescribing: a systematic review and meta-analysis. *JAMA Netw Open.* 2018;1(6):e183750. doi:10.1001/jamanetworkopen.2018.3750

eTable 1. Example Search Strategy (PsycINFO, American Psychological Association)

eTable 2. Summary of Included Studies

eTable 3. Economic Measures and Outcomes of Medication Review

eReferences

This supplementary material has been provided by the authors to give readers additional information about their work.

© 2018 Sheehan R et al. *JAMA Network Open.*

eTable 1. Example Search Strategy (PsycINFO, American Psychological Association)

	Search term and limits
1	(medication* adj2 review*).ti,ab
2	(drug* adj2 review*).ti,ab.
3	(prescri* adj2 review*).ti,ab
4	(medication* adj2 monitoring).ti,ab
5	(drug* adj2 monitoring).ti,ab
6	(prescri* adj2 monitoring).ti,ab
7	(medication* adj2 optimi#ation).ti,ab
8	(drug* adj2 optimi#ation).ti,ab
9	(prescri* adj2 optimi#ation).ti,ab
10	1 OR 2 OR 3 OR 4 OR 5 OR 6 OR 7 OR 8 OR 9
11	psychotropic*.ti,ab
12	Anti?psychotic*.ti,ab
13	neuroleptic*.ti,ab
14	major tranquil?i#er*.ti,ab
15	mood stabili#er*.ti,ab
16	anti?depressant*.ti,ab
17	sedative*.ti,ab
18	hypnotic*.ti,ab
19	anxiolytic*.ti,ab
20	minor tranquil?i#er*.ti,ab
21	“neuroleptic drugs”/
22	“mood stabilizers”/
23	“antidepressant drugs”/
24	“hypnotic drugs”/
25	“sedatives”/
26	11 OR 12 OR 13 OR 14 OR 15 OR 16 OR 17 OR 18 OR 19 OR 20 OR 21 OR 22 OR 23 OR 24 OR 25
27	(psychotropic* adj2 review*).ti,ab
28	(anti?psychotic* adj2 review*).ti,ab
29	(neuroleptic* adj2 review*).ti,ab
30	(major tranquil?i#er* adj2 review*).ti,ab
31	(mood stabili#er* adj2 review*).ti,ab
32	(anti?depressant* adj2 review*).ti,ab
33	(sedative* adj2 review*).ti,ab
34	(hypnotic* adj2 review*).ti,ab
35	(anxiolytic* adj2 review*).ti,ab
36	(minor tranquil?i#er* adj2 review*).ti,ab
37	27 OR 28 OR 29 OR 30 OR 31 OR 32 OR 33 OR 34 OR 35 OR 36
38	(psychotropic* adj2 optimi#ation).ti,ab
39	(anti?psychotic* adj2 optimi#ation).ti,ab
40	(neuroleptic* adj2 optimi#ation).ti,ab
41	(major tranquil?i#er* adj2 optimi#ation).ti,ab
42	(mood stabili#er* adj2 optimi#ation).ti,ab
43	(anti?depressant* adj2 optimi#ation).ti,ab
44	(sedative* adj2 optimi#ation).ti,ab

© 2018 Sheehan R et al. *JAMA Network Open.*

	Search term and limits
45	(hypnotic* adj2 optimi#ation).ti,ab
46	(anxiolytic* adj2 optimi#ation).ti,ab
47	(minor tranquil?i#er* adj2 optimi#ation).ti,ab
48	38 OR 39 OR 40 OR 41 OR 41 OR 42 OR 43 OR 44 OR 45 OR 46 OR 47
49	(psychotropic* adj2 monitoring).ti,ab
50	(anti?psychotic* adj2 monitoring).ti,ab
51	(neuroleptic* adj2 monitoring).ti,ab
52	(major tranquil?i#er* adj2 monitoring).ti,ab
53	(mood stabili#er* adj2 monitoring).ti,ab
54	(anti?depressant* adj2 monitoring).ti,ab
55	(sedative* adj2 monitoring).ti,ab
56	(hypnotic* adj2 monitoring).ti,ab
57	(anxiolytic* adj2 monitoring).ti,ab
58	(minor tranquil?i#er* adj2 monitoring).ti,ab
59	49 OR 50 OR 51 OR 52 OR 53 OR 54 OR 55 OR 56 OR 57 OR 58 OR 59
60	37 OR 48 OR 59
61	10 AND 26
62	60 OR 61

© 2018 Sheehan R et al. *JAMA Network Open.*

eTable 2. Summary of Included Studies

Randomized controlled trials

**Ballard et al,[1] 2016**	
Study title	Impact of antipsychotic review and nonpharmacological intervention on antipsychotic use, neuropsychiatric symptoms, and mortality in people with dementia living in nursing homes: a factorial cluster-randomized controlled trial by the well-being and health for older people with dementia (WHELD) program
Study design	Factorial cluster-randomized controlled trial
Location	UK
Setting	Sixteen nursing homes
Number of participants	*n*=277
Participant characteristics	People with dementia (12% mild, 40% moderate, 47% severe)<br>Mean (SD) age 85.3 (7.02) years<br>74% female<br>18% prescribed antipsychotic drugs
Intervention	Multi-modal (impact of medication review reported separately); all nursing homes received training in person-centred care and participants were entered into a trial with a 2x2x2 factorial design with a combination of, antipsychotic drug review, social interaction intervention, and an exercise intervention. Antipsychotic review based on guidelines from NICE[a], Alzheimer's Society, and the UK Department of Health and delivered by participants' usual physician (general practitioner or psychiatrist). Selected staff in each home were encouraged to prompt medication review in those suitable.
Duration of follow-up	9 months
Optimisation outcome measures and results	**Proportion of participants prescribed antipsychotic drugs**<br>Significantly lower proportion prescribed antipsychotic drugs in the medication review group compared with the non-review group at follow-up (adjusted OR[b] 0.17, 95% CI 0.05 to 0.60, p=0.006).<br>**Neuropsychiatric symptoms (measured with NPI[c])**<br>Those receiving medication review had a 7.37 point (95% CI 1.53 to 13.22, p=0.02) disadvantage in NPI at follow-up compared with those not receiving review<br>**Agitation (Cohen-Mansfield Agitation Inventory)**<br>No difference between intervention and control groups (adjusted score difference 4.60, 95% CI -1.43 to 10.63, p=0.13)<br>**Mortality**<br>Antipsychotic review conferred a non-significant reduction in mortality compared with the group not receiving review (OR 0.67, 95% CI 0.39 to 1.14, p=0.15)
Author's conclusions	**Reductions in antipsychotic drug prescribing can be achieved but may not benefit people with dementia**
Source of funding	UK National Institute for Health Research
Quality assessment	Good
Critical appraisal	Lack of blinding<br>Intervention varied or poorly described<br>Control condition not well described<br>High attrition<br>Multiple testing

[a]NICE, National Institute for Health and Care Excellence; [b]OR, odds ratio; [c]NPI, neuro-psychiatric inventory

**Ballard et al,[2] 2017**

© 2018 Sheehan R et al. *JAMA Network Open.*

Study title	Impact of antipsychotic review and non-pharmacological intervention on health-related quality of lie in people with dementia living in care homes: WHELD – a factorial cluster randomised controlled trial
Study design	Factorial cluster-randomized controlled trial (reports secondary outcome of Ballard et al, 2016a)
Location	UK
Setting	Sixteen nursing homes
Number of participants	*n*=277
Participant characteristics	People with dementia (12% mild, 40% moderate, 47% severe)<br>Mean (SD) age 85.3 (7.02) years<br>74% female<br>18% prescribed antipsychotic drugs
Intervention	Multi-modal (impact of medication review reported separately); all nursing homes received training in person-centered care and were then randomized to a combination of, antipsychotic drug review, social interaction intervention, and an exercise intervention. Antipsychotic review based on guidelines from NICE[a], Alzheimer's Society, and the UK Department of Health and delivered by participants' usual physician (general practitioner or psychiatrist). Selected staff in each home were encouraged to prompt medication review in those suitable.
Duration of follow-up	9 months
Optimisation outcome measures and results	**Health-related quality of life (measured with DEMQOL-proxy)**<br>Those receiving medication review showed a 4.54 point (95% CI -9.26 to 0.19, p=0.06) worsening in their DEMQOL-proxy score at follow-up compared with those in control group
Author's conclusions	Medication review and subsequent antipsychotic discontinuation had a detrimental impact on health-related quality of life
Source of funding	National Institute for Health Research
Quality assessment	Good
Critical appraisal	Lack of blinding<br>Intervention varied or poorly described<br>Control condition not well described<br>High attrition<br>Multiple testing

[a]NICE, National Institute for Health and Care Excellence

**Jordan et al,[3] 2015**	
Study title	Nurse-led medicines' monitoring for patients with dementia in care homes
Study design	Pragmatic cohort stepped wedge cluster randomized trial
Location	UK
Setting	Five nursing homes
Number of participants	*n*=41
Participant characteristics	People with dementia prescribed a antipsychotic, anti-depressant, or anti-epileptic medication<br>Mean (SD) age 78.7 (11.0) years<br>61% female
Intervention	Monthly nurse-led medication review by application of West Wales Adverse Drug Reaction Profile for Mental Health Medicines
Duration of follow-up	5 months
Optimisation outcome measures and results	**Number of drug-related problems identified**<br>More problems were identified when the Profile was used compared with not used (adjusted mean difference 9.06, 95%CI[a] 7.95 to 10.16)<br>**Actions to address drug-related problems identified**<br>Problems were actioned more frequently when the Profile was used compared with not used (adjusted mean difference 3.34, 95%CI 2.57 to 4.11)<br>**Changes in psychotropic medication**

	Reductions in mental health medicines were more likely when the Profile was used compared with not used (adjusted OR[b] 4.45, 95% CI 1.15 to 17.22)<br>**Functional status (measured with Bristol ADL[c] scale)**<br>No significant difference between intervention and control (mean difference between groups 0.45, 95%CI -0.47 to 0.93, p=0.52)<br>**Dementia psychopathology (measured with MOUSEPAD[d])**<br>No significant difference between intervention and control (mean difference between groups 4.67, 95%CI -0.04 to 2.78, p=0.06)
Author's conclusions	Nurse-led medication review using the WWADR increased the numbers of drug-related problems identified and addressed and reduced prescriptions of psychotropic drugs. The intervention is feasible, low cost, low risk, and convenient.
Source of funding	Wales School for Primary Care Research
Quality assessment	Fair
Critical appraisal	Selection bias<br>Lack of blinding<br>Use of unvalidated measures / non-standard assessment tools<br>Small sample<br>Intervention varied or poorly described<br>Multiple testing

[a]CI, confidence interval; [b]OR, odds ratio; [c]ADL, activities of daily living; [d]MOUSEPAD, Manchester and Oxford Universities Scale for the Psychopathological Assessment of Dementia

**Moncrieff et al,[4] 2016**	
Study title	Results of a pilot cluster randomised trial of the use of a medication review tool for people taking antipsychotic medication
Study design	Cluster randomized controlled trial (pilot study)
Location	UK
Setting	Community psychiatric teams
Number of participants	*n*=60
Participant characteristics	Adults with a psychotic disorder taking antipsychotic medication<br>Mean age 42.1 years<br>72% male
Intervention	A Medication Review Tool to be completed by participants (with support, if needed) prior to a psychiatrist appointment to guide discussion about antipsychotic medication
Duration of follow-up	3 months
Optimisation outcome measures and results	**Decision Self-Efficacy Scale**<br>No significant difference between intervention and control group (adjusted difference -4.16, 95%CI -9.81 to 1.49)<br>**Client Satisfaction Questionnaire**<br>No significant difference between intervention and control group (mean score 27 in intervention group, 28 in control group, p>0.05)<br>**Drug Attitude Inventory**<br>No significant difference between intervention and control groups (adjusted difference 1.65, 95%CI -0.09 to 3.40)<br>**Liverpool University Neuroleptic Side-Effect Rating Scale**<br>No significant difference between intervention and control groups (adjusted difference -0.42, 95%CI -8.12 to 7.29)<br>**Brief Positive and Negative Syndrome Scale**<br>No significant difference between intervention and control groups (adjusted difference 0.13, 95%CI -2.21 to 2.48)<br>**Medication Adherence Questionnaire**<br>Participants in the intervention group had a lower score indicating greater propensity to be adherent with medication (adjusted difference -0.44, 95%CI -0.76 to -0.11)<br>**Change in antipsychotic medication[b]**<br>No significant difference between intervention and control groups (OR[b] 2.64, 95% CI 0.84, 8.31)

© 2018 Sheehan R et al. *JAMA Network Open.*

	**Antipsychotic polypharmacy (≥2 antipsychotics prescribed)**<br>No difference between intervention and control groups
Author's conclusions	The Medication Review Tool did not improve participants' confidence in decisions about their antipsychotic medication
Source of funding	National Institute for Health Research
Quality assessment	Good
Critical appraisal	Selection bias<br>Baseline differences between intervention and control group<br>Lack of blinding<br>Intervention varied or poorly described<br>Multiple testing<br>Possible contamination of control group<br>Pilot study not powered to detect difference in outcome measures

[a]Additional data provided by authors; [b]OR, odds ratio

**Patterson et al,[5] 2010**	
Study title	An evaluation of an adapted US model of pharmaceutical care to improve psychoactive prescribing for nursing home residents in Northern Ireland (Fleetwood Northern Ireland Study)
Study design	Cluster randomized controlled trial
Location	UK
Setting	Twenty two nursing homes
Number of participants	*n*=334
Participant characteristics	Older adults<br>Mean (SD) age 82 (8.4) years<br>73% female<br>68% moderately-severely cognitively impaired<br>71% prescribed psychotropic medication
Intervention	Face-to-face monthly medication review by study pharmacist. Algorithm was used to indicate inappropriate psychotropic medication. Pharmacist recommendations for medication changes discussed with General Practitioner.
Duration of follow-up	12 months
Optimisation outcome measures and results	**Proportion of nursing home residents prescribed ≥1 inappropriate psychotropic drug at follow-up**<br>Significantly lower proportion prescribed inappropriate psychotropic drugs in intervention group compared with the control group at follow-up (intervention group proportion: 20%, control group proportion: 50%, $p<0.001$; adjusted OR[a] for receiving inappropriate psychotropic medication 0.26 (0.14 to 0.49))<br>**Rate of falls**<br>No significant difference in rate of falls between intervention and control groups (intervention group rate: 16.3 falls per 100 person-months, control group rate: 11.4 falls per 100 person-months, $p=0.09$)
Author's conclusions	A pharmacist-delivered program of medication review targeting specific drugs can result in marked reduction in inappropriate psychotropic drug prescribing but had no effect on falls
Source of funding	Health and Social Care Research and Development Office<br>Health and Social Services Boards, Northern Ireland
Quality assessment	Fair
Critical appraisal	Baseline differences between intervention and control group<br>Lack of blinding<br>Use of unvalidated measures / non-standard assessment tools (secondary outcome)<br>High attrition

[a]OR, odds ratio

Before-after studies

**Bach et al,[6] 2017**

Study title	Improving nursing home compliance via revised antipsychotic use survey tool
Study design	Before-after
Location	USA
Setting	Two nursing homes
Number of participants	*n*=20
Participant characteristics	People with dementia (and without a psychotic or mood disorder) prescribed antipsychotic medication for behavioral and psychological symptoms<br>Mean (SD) age 87.1 (7.9) years<br>90% female
Intervention	Monthly medication reviews by a pharmacist using a modified version of the CDPH[a] antipsychotic use tool designed to improve compliance with prescribing guidelines. Pharmacist made recommendations to the prescribing physician who made treatment decisions
Duration of follow-up	7 months
Optimisation outcome measures and results	**Number (proportion) of participants with antipsychotic drug discontinuation after medication review**<br>4/20 (20%)
Author's conclusions	Using a survey tool with pharmacist medication review may help improve quality of prescribing of antipsychotic drugs and reduce antipsychotic prescribing in people with dementia
Source of funding	Not given
Quality assessment	Fair
Critical appraisal	Selection bias<br>Lack of blinding<br>Small sample<br>Lack of control group<br>Statistical tests inappropriate or missing<br>Lack of clinical outcomes

[a]CDPH, California Department of Public Health

**Bisconer et al,[7] 1995**	
Study title	Impact of a psychotropic medication and physical restraint review process on adults with mental retardation, psychiatric diagnoses, and challenging behaviors
Study design	Before-after
Location	USA
Setting	Intermediate care facility for adults with intellectual disability
Number of participants	*n*=80
Participant characteristics	People with intellectual disability (22.5% mild-moderate, 77.5% severe-profound)<br>Mean age (range) 34.5 (20-61) years<br>75% male<br>40% had axis I mental disorder
Intervention	A multi-disciplinary team reviewed the use of psychotropic medication. Reviews include a standard information set including medical and drug history followed by open discussion. Recommendations are made. Cases are reviewed every six months. Review team included staff and lay members from outside the organisation.
Duration of follow-up	Mean 2.5 years
Optimisation outcome measures and results	**Total number of prescriptions for psychotropic medications**<br>Reduction from 149 prescriptions at baseline to 84 prescriptions at follow-up<br>**Average number of psychotropic prescriptions per person**<br>Reduction from 1.86 at baseline to 1.05 at follow-up<br>**Proportion of participants with change in psychotropic drugs**<br>47/80 (59%) changed psychotropic medication<br>**Antipsychotic polypharmacy**

	Reduction from 4 (5%) participants at baseline to 0 (0%) participants at follow up<br>**Reported medication side-effects**<br>Side-effects reported in 11 (14%) participants at baseline and 8 (10%) participants at follow-up<br>**Challenging behavior frequency counts**<br>Reduction in the mean number of challenging behavior incidents per month
Author's conclusions	The review process led to improvements in data gathering and documentation and reductions in the use of psychotropic medication use without accompanying increase in challenging behavior
Source of funding	Not given
Quality assessment	Fair
Critical appraisal	Selection bias<br>Lack of blinding<br>Use of unvalidated measures / non-standard assessment tools<br>Statistical tests inappropriate or missing<br>No control group

**Branford,[8] 1996**	
Study title	A review of antipsychotic drugs prescribed for people with learning disabilities who live in Leicestershire
Study design	Before-after
Location	UK
Setting	Hospitals, hostels, and community group homes
Number of participants	*n*=198
Participant characteristics	People with intellectual disability (44% mild-moderate, 56% severe-profound)<br>Mean (SD) age 43 (12.3) years<br>66% male<br>A minority had diagnosed mental illness
Intervention	Case reviews including medication history, standardized assessments of behavior (ABC[a]) and psychopathology (Reiss screen and PIMRA[b]), used to inform multi-disciplinary discussion (pharmacist, psychiatrist, and nursing staff). Medication decisions were made according to a flexible protocol
Duration of follow-up	1 year
Optimisation outcome measures and results	**Proportion reducing antipsychotic medication after initial medication review**<br>123/198 (62%) underwent dose reduction<br>**Antipsychotic dose at follow-up (those undergoing antipsychotic reduction after initial review)**<br>31/123 (25%) discontinued antipsychotic medication<br>56/123 (46%) reduced dose<br>27/123 (22%) same dose<br>9/123 (7%) higher dose<br>**Clinical presentation of those undergoing antipsychotic reduction after initial review**<br>31/123 (25%) undergoing medication reduction after initial review had a "good" outcome<br>52/123 (42%) had a "poor" outcome<br>40 /123 (33%) had "unclear" outcome
Author's conclusions	Antipsychotic drug review program resulted in a withdrawal or sustained reduction in dose at 12 months in just under half those reviewed. However a relatively high proportion of case review recommendations to reduce or withdraw medication failed
Source of funding	Not stated
Quality assessment	Fair
Critical appraisal	Lack of blinding

© 2018 Sheehan R et al. *JAMA Network Open.*

	Use of unvalidated measures / non-standard assessment tools<br>Statistical tests inappropriate or missing<br>No control group<br>Selective reporting / incomplete outcome data<br>Lack of clinical outcomes

[a]ABC, Aberrant Behavior Checklist; [b]PIMRA, Psychopathology Instrument for Mentally Retarded Adults

**Child et al,[9] 2012**	
Study title	A pharmacy led program to review anti-psychotic prescribing for people with dementia
Study design	Before-after
Location	UK
Setting	Primary care<br>Participants living in care homes or in family home
Number of participants	*n*=70
Participant characteristics	Adults with dementia on long-term low-dose antipsychotic drugs prescribed in primary care
Intervention	Antipsychotic drug review by a specialist pharmacist who had access to clinical and care home notes and liaised with care staff and the General Practitioner. Consensus decision reached about antipsychotic withdrawal attempts.
Duration of follow-up	No follow-up – outcome measured immediately after intervention
Optimisation outcome measures and results	**Proportion with antipsychotic drug dose reduction or discontinuation**<br>Antipsychotic drug withdrawn or dose reduced in 43/70 (61.4%) cases
Author's conclusions	Pharmacist-led review can successfully limit the prescribing of antipsychotics to people with dementia
Source of funding	Not given
Quality assessment	Fair
Critical appraisal	Lack of blinding<br>Statistical tests inappropriate or missing<br>Missing baseline information<br>Lack of control group<br>Lack of clinical outcome measure<br>Short follow-up

**Craig et al,[10] 1984**	
Study title	Clinician-computer interaction: automated review of psychotropic drugs
Study design	Before-after
Location	USA
Setting	Psychiatric hospital
Number of participants	Not given
Participant characteristics	Adults receiving psychotropic medication<br>Further details not given
Intervention	Two-stage review; automated (computerized) medication review to flag high doses and polypharmacy (drug exceptions) followed by a targeted review by the patient's treating psychiatrist and clinical supervisor
Duration of follow-up	No follow-up – outcome measured immediately after intervention
Optimisation outcome measures and results	**Number (proportion) of review of drug exceptions resulting in medication change**<br>67/263 (25.5%) drug exceptions identified in one year resulted in a change after clinician medication review
Author's conclusions	An automated system is an efficient system to highlight potentially inappropriate prescribing and direct medication reviews that regularly result in corrective actions
Source of funding	Not given

© 2018 Sheehan R et al. *JAMA Network Open.*

Quality assessment	Poor
Critical appraisal	Lack of blinding<br>Statistical tests inappropriate or missing<br>Missing baseline information<br>Intervention varied or poorly described<br>No control group<br>Lack of clinical outcome measure<br>Short follow-up

**Dahl et al,[11] 2008**	
Study title	Quality improvement in long-term care: the Psychotropic Assessment Tool (PAT)
Study design	Before-after
Location	USA
Setting	Private long-term care facility (care home)
Number of participants	n=110
Participant characteristics	People with dementia (mean (SD) MMSE[a] 13.5 (7.3))<br>Mean (SD) age 83.8 (7.5) years<br>74% female
Intervention	Implementation of a medication review program guided by a Psychotropic Assessment Tool (PAT) to highlight psychiatric/behavioral symptoms, side-effects of psychotropic drugs, and prompt patient/carer input. The PAT was completed six-monthly by a nurse, senior nurse, social worker, and family member, and discussed in interdisciplinary review meetings consisting of a pharmacist, physician, manager, and social worker. This team make recommendations for psychotropic treatment to the prescribing physician.
Duration of follow-up	12 months
Optimisation outcome measures and results	**Proportion of residents prescribed psychotropic drug, by type, pre- and post-intervention**<br>Antipsychotics 26.5% (pre) and 25.2% (post)<br>Anti-depressants 55.8% (pre) and 55.7% (post)<br>Anxiolytics 6% (pre) and 4% (post)<br>Hypnotics 2.6% (pre) and 3.4% (post)<br>Acetylcholinesterase inhibitors 69% (pre) and 61.7% (post)<br>Memantine 51% (pre) and 52.2% (post)
Author’s conclusions	The medication review using the PAT did not change medication use significantly but resulted in improved communication and information exchange within and between the multi-disciplinary team and families
Source of funding	Not given
Quality assessment	Fair
Critical appraisal	Lack of blinding<br>No control group<br>Statistical tests inappropriate or mission<br>No control group<br>Not a fixed cohort<br>Lack of clinical outcome measure

[a]MMSE, mini mental state examination

**Donat,[12] 2006**	
Study title	Impact of a clinical-administrative review procedure on reducing reliance on psychotropic PRN medication
Study design	Before-after
Location	USA
Setting	Psychiatric hospital
Number of participants	Not given
Participant characteristics	Not given

© 2018 Sheehan R et al. *JAMA Network Open.*

Intervention	Two stage review; automated medication review to flag excessive use of PRN medication (≥ 3 uses per week) followed by targeted review by local psychologist and psychiatrist. Persistent PRN psychotropic use of ≥3 uses per week mandates senior clinician team medication review
Duration of follow-up	12 months
Optimisation outcome measures and results	**Average number of patients receiving ≥ 3 PRN psychotropic medications per week pre- and post-intervention**<br>20.8 individuals per week exceeded PRN threshold before implementation of the medication review system, and average of 12.4 individuals after medication review implemented (statistically significant trend)
Author's conclusions	The medication review procedure can have a major impact on the reliance on PRN medication
Source of funding	Not given
Quality assessment	Fair
Critical appraisal	Lack of blinding<br>Missing baseline information<br>Intervention varied or poorly described<br>No control group<br>Lack of clinical outcome measure<br>Not a fixed cohort

**Ellenor and Frisk,[13] 1977**	
Study title	Pharmacist impact on drug use in an institution for the mentally retarded
Study design	Before-after
Location	USA
Setting	Institution for people with intellectual disability
Number of participants	*n*=208
Participant characteristics	Incompletely described
Intervention	Pharmacist psychotropic medication review with recommendations to a multi-disciplinary review committee
Duration of follow-up	Maximum 2 years
Optimisation outcome measures and results	**Overall change in use of psychotropic medications**<br>Reduction in number of medications from 496 pre-intervention to 313 post-intervention, from average of 2.4 to 1.5 per person<br>**Change in use of psychotropic medications, by type**<br>Antipsychotics: 234 prescriptions pre-intervention – 83 discontinued, 101 dose reduction, 65 higher dose/initiation, 27 no change<br>Anti-depressants: 160 prescriptions pre-intervention – 80 discontinued, 18 dose reduction, 4 higher dose/initiation, 58 no change<br>Sedative-hypnotics: 85 prescriptions pre-intervention – 64 discontinued, 5 dose reduction, 14 higher dose/initiation, 17 no change<br>**Drug expenditure**<br>Savings related to altered drug therapies estimated at $10,000/year (1977)<br>**Change in challenging behavior (measured with ABC[a])**<br>"Slight increase" in ABC in discontinuation, dose reduction, and a comparison group not part of the medication review program, no significant difference between groups undergoing review and those not reviewed
Author's conclusions	Reductions in drug use possible with pharmacy and team approach, without significant clinical deteriorations
Source of funding	Not given
Quality assessment	Poor

© 2018 Sheehan R et al. *JAMA Network Open.*

Critical appraisal	Selection bias<br>Lack of blinding<br>Statistical tests inappropriate or missing<br>Missing baseline information<br>No control group<br>Selective reporting / incomplete outcome data

[a]ABC, Adaptive Behavioral Scale

**Ferguson et al,[14] 1982**	
Study title	Effects of data-based interdisciplinary medication reviews on the prevalence and pattern of neuroleptic drug use with institutionalized mentally retarded persons
Study design	Before-after
Location	USA
Setting	Institution for people with intellectual disability
Number of participants	*n*=97
Participant characteristics	People with intellectual disability (most with severe-profound degree)<br>Age range 14 to 70 years<br>Prescribed antipsychotic drugs for challenging behavior
Intervention	A multi-disciplinary team who met each month to review presentation and antipsychotic medication. Review included discussion of benefits and side-effects of antipsychotics. Guidelines for antipsychotic medication reduction were applied
Duration of follow-up	Up to 18 months
Optimisation outcome measures and results	**Number (proportion) of participants discontinuing antipsychotic medication**<br>Reduced from 97 before intervention to 29 after intervention (70%)
Author's conclusions	A medication review program can play an important role in the monitoring and regulation of drug use in an institution
Source of funding	National Institute of Mental Health
Quality assessment	Fair
Critical appraisal	Selection bias<br>Lack of blinding<br>Statistical tests inappropriate or missing

**Gallimore et al,[15] 2016**	
Study title	Pharmacist medication reviews to improve safety monitoring in primary care patients
Study design	Before-after
Location	USA
Setting	Psychiatric out-patient clinic
Number of participants	*n*=144
Participant characteristics	People accessing psychiatric out-patient care<br>Mean (range) age 42.6 (11-51) years<br>62% female<br>Average 3.2 mental health diagnoses per patient<br>73% ≥2 psychotropic medications
Intervention	Notes-based antipsychotic or mood stabilizer medication review by pharmacist 1-3 months after a psychiatry consultation which included review of the electronic health record for evidence of drug monitoring and potential drug interactions. Recommendations were submitted to the general practitioner
Duration of follow-up	3 months
Optimisation outcome measures and results	**Proportion with up-to-date monitoring (laboratory and other measures in accordance with national consensus guidelines)**<br>54.1% before medication review, 72.1% after medication review (p=0.0001)<br>**Proportion with up-to-date assessment of movement side-effects (AIMS[a]) in those prescribed antipsychotic drugs**

	75.0% before medication review, 63.5% after medication review (p=0.2113)<br>**Proportion at risk of drug-drug interaction**<br>43.8% before medication review, 24.3% after medication review (p<0.0001)
Author's conclusions	Pharmacist medication reviews are associated with significant increase in patients receiving guideline-recommended monitoring and reduction in patients at risk for drug-drug interactions
Source of funding	Not given
Quality assessment	Good
Critical appraisal	Selection bias<br>Lack of blinding<br>Lack of control group<br>Lack of clinical outcome measure

[a]AIMS, abnormal involuntary movement scale

**Gemelli et al,[16] 2016**	
Study title	Evaluating the impact of pharmacists on reducing use of sedative / hypnotics for treatment of insomnia in long-term care facility residents
Study design	Before-after
Location	USA
Setting	Care homes
Number of participants	*n*=34
Participant characteristics	Residents of elderly care homes prescribed sedative / hypnotic medication for insomnia<br>Mean (range) age 80.8 (68-94) years<br>69% female
Intervention	Pharmacist chart review with recommendations and advice sent to prescriber (physician)
Duration of follow-up	3 months
Optimisation outcome measures and results	**Number (proportion) of participants with reduction or discontinuation in sedative/hypnotic drug following review**<br>(At least) 16/36 (48%) participants underwent reduction or discontinuation after review
Author's conclusions	Pharmacist intervention can have a meaningful impact on reducing inappropriate sedative / hypnotic use in the elderly population
Source of funding	No funding received
Quality assessment	Fair
Critical appraisal	Lack of blinding<br>Small sample<br>Statistical tests inappropriate or missing<br>Lack of control group<br>Lack of clinical outcome measure<br>High attrition (>20% drop out)

**Glaser & Morreau,[17] 1986**	
Study title	Effects of interdisciplinary team review on the use of antipsychotic agents with severely and profoundly mentally retarded persons
Study design	Before-after
Location	USA
Setting	Institution for people with intellectual disability
Number of participants	*n*=28
Participant characteristics	People with intellectual disability (severe-profound) receiving antipsychotic drugs for challenging behavior<br>71% female
Intervention	Monthly multi-disciplinary antipsychotic medication review meetings
Duration of follow-up	6 months

Optimisation outcome measures and results	**Number (proportion) with change to antipsychotic drug before (without) and after (with) intervention**<br>Greater number of participants underwent change in antipsychotic drugs occurred with the intervention (17/28) than without the intervention (11/28)<br>**Total dose of antipsychotic drugs prescribed before (without) and after (with) intervention**<br>Decrease in total dose of antipsychotic dose used with intervention<br>**Aggressive challenging behavior**<br>No difference in number of incidents of aggressive challenging behavior in the group undergoing intervention compared with groups not undergoing intervention
Author's conclusions	Multi-disciplinary medication reviews can result in reductions in the prescription of antipsychotic drugs used for challenging behavior and is not associated with worsening of aggression
Source of funding	Not given
Quality assessment	Good
Critical appraisal	Lack of blinding<br>Use of unvalidated measures / non-standard assessment tools<br>Small sample size<br>No control group

**Inoue,[18] 1982**	
Study title	A clinical pharmacy service to reduce psychotropic medication use in an institution for mentally handicapped persons
Study design	Before-after
Location	Canada
Setting	Institution for people with intellectual disability
Number of participants	*n*=251
Participant characteristics	Incompletely reported
Intervention	Pharmacist medication review including evaluation of drug response, side-effects, and suggesting alterations to the drug regimen using a standard template used to inform a monthly multi-disciplinary medication review meeting
Duration of follow-up	6 months
Optimisation outcome measures and results	**Changes in medications following medication review**<br>Discontinuation: 135/272 medications<br>Dose reduction: 91/272 medications<br>Change of medication: 24/272 medications<br>Dose increase: 7/272 medications<br>Overall 257/272 medications were changed<br>**Changes in number of psychotropic drug prescriptions before and after intervention, by type**<br>Antipsychotics: 181 prescriptions before intervention, 119 prescriptions after intervention<br>Anti-depressants: 22 prescriptions before intervention, 4 prescriptions after intervention<br>Anxiolytics: 40 prescriptions before intervention, 6 prescriptions after intervention<br>Sedatives / hypnotics: 27 prescriptions before intervention, 5 prescriptions after intervention<br>Lithium: 2 prescriptions before intervention, 3 prescriptions after intervention<br>**Number (proportion) of participants discontinuing psychotropic medication**<br>121 (48%) participants discontinued psychotropic medication following medication review intervention<br>**Clinical result of drug changes (subjective assessment)**<br>Positive change: 248/257 (96.5%)

© 2018 Sheehan R et al. *JAMA Network Open.*

	Negative change 9/257 (3.5%)
Author's conclusions	The pharmacist medication review made considerable impact on psychotropic medication use in a population of intellectually disabled people
Source of funding	Not given
Quality assessment	Fair
Critical appraisal	Lack of blinding<br>Use of unvalidated measures / non-standard assessment tools<br>Statistical tests inappropriate or missing<br>Missing baseline information<br>No control group

**Jauernig et al,[19] 1995**	
Study title	Evaluation of an interdisciplinary review committee managing the use of psychotropic medication with people with intellectual disabilities
Study design	Before-after
Location	Australia
Setting	Institution for people with intellectual disability
Number of participants	*n*=25
Participant characteristics	Incompletely reported
Intervention	Multi-disciplinary team conduct medication review according to a standard structure and provided recommendations to responsible physician. Rolling process – average of 12 reviews per participant.
Duration of follow-up	2 years
Optimisation outcome measures and results	**Number (proportion) of participants with change to psychotropic medication**<br>19/25 (76%) reduced dose<br>3/25 (12%) discontinued<br>0/25 (0%) increased dose<br>**Number (proportion) of participants receiving psychotropic polypharmacy**<br>Psychotropic polypharmacy: 13 (52%) before intervention, 6 (24%) at follow-up<br>**Change in challenging behavior (frequency counts)**<br>Average daily frequency of challenging behavior lower at the end of the program than at baseline for 20/25 participants (full data not reported)
Author's conclusions	Multi-disciplinary medication review is effective in reducing use of psychotropic medication in people with intellectual disabilities in a residential setting
Source of funding	Not given
Quality assessment	Poor
Critical appraisal	Selection bias<br>Lack of blinding<br>Use of unvalidated measures / non-standard assessment tools<br>Small sample<br>Statistical tests inappropriate or missing<br>Missing baseline information

**Johnson et al,[20] 2012**	
Study title	Reviewing long-term anti-depressants can reduce drug burden
Study design	Before-after
Location	UK
Setting	Primary care
Number of participants	*n*=2,849
Participant characteristics	Long term (≥2 year) adult users of anti-depressants, not under secondary care and without severe mental illness

© 2018 Sheehan R et al. *JAMA Network Open.*

	Mean (SD) age 52.4 (13.4) years<br>73.4% female
Intervention	General practitioner face-to-face medication review
Duration of follow-up	No follow-up – outcome measured immediately after intervention
Optimisation outcome measures and results	**Number (proportion) of participants with change in anti-depressant treatment after medication review**<br>811 (28.5%) had any change in treatment<br>199 (7.0%) stopped anti-depressant<br>366 (12.8%) reduced anti-depressant dose<br>150 (5.3%) increased anti-depressant dose<br>96 (3.4%) switched anti-depressant drug<br>**Group average change in anti-depressant prescribed daily dose**<br>9.5% (95%CI 9.1% to 9.8%, p<0.001) reduction in mean daily dose
Author's conclusions	Appropriate reductions in anti-depressant prescribing can be achieved by general practitioner review
Source of funding	Partly by National Health Service (via incentive payments to practices undertaking medication reviews)
Quality assessment	Fair
Critical appraisal	Selection bias<br>Lack of blinding<br>Missing baseline information<br>Intervention varied or poorly described<br>Lack of control group<br>Lack of clinical outcome measure<br>Short follow-up

**Laska et al,[21] 1980**	
Study title	Automated review system for orders of psychotropic drugs
Study design	Before-after
Location	USA
Setting	Psychiatric hospital
Number of participants	Not reported
Participant characteristics	Not reported
Intervention	Two stage review; automated medication review to flag 'drug exceptions' (drug interactions or inappropriate doses) followed by clinical medication review by treating physician and clinical supervisor, and drug review committee, if necessary
Duration of follow-up	Study period 3 years
Optimisation outcome measures and results	**Average number of 'drug exceptions' (polypharmacy or dose-range exceptions) per patient over time**<br>Decrease from 0.34 exceptions / patient to 0.10 exceptions / patient over study period
Author's conclusions	The intervention was associated with a substantial reduction in prescribing in breach of clinical guidelines
Source of funding	Not given
Quality assessment	Fair
Critical appraisal	Lack of blinding<br>Missing baseline information<br>Intervention varied or poorly described<br>No control group<br>Lack of clinical outcome measure<br>Not a fixed cohort

**Lepler et al,[22] 1993**	
Study title	Implementation of an inter-disciplinary psychotropic drug review process for community-based facilities
Study design	Before-after

© 2018 Sheehan R et al. *JAMA Network Open.*

Location	USA
Setting	Community intellectual disability provider
Number of participants	*n*=12
Participant characteristics	Incompletely reported
Intervention	Three-monthly multi-disciplinary team review of psychotropic medications on ongoing basis to discuss medication effectiveness, side-effects, and other interventions. Consensus recommendations are made and responsible physician is informed
Duration of follow-up	Up to 4 years
Optimisation outcome measures and results	**Number (proportion) of participants with reduced dose of psychotropic medication**<br>9 (75%) individuals maintained on lower psychotropic dose after the intervention
Author's conclusions	Using a systematic method of medication review has enabled adjustments to psychotropic medication regimens leading to optimal functioning and mental health
Source of funding	Not given
Quality assessment	Poor
Critical appraisal	Lack of blinding<br>Small sample<br>Statistical tests inappropriate or missing<br>Missing baseline information<br>No control group<br>Lack of clinical outcome measure

**Marcoux,[23] 1985**	
Study title	Implementation of a psychotropic drug review service in a mental retardation facility
Study design	Before-after
Location	USA
Setting	Institution for people with intellectual disability
Number of participants	*n*=255
Participant characteristics	Not reported
Intervention	Structured multi-disciplinary team review of psychotropic medication supported by standard data collection forms
Duration of follow-up	10 months
Optimisation outcome measures and results	**Group average change in antipsychotic drug use**<br>Decrease of 17% in average dose of antipsychotic drugs
Author's conclusions	The psychotropic medication review service has been successful in generating cost savings for the institution and an educational process for staff
Source of funding	Not given
Quality assessment	Poor
Critical appraisal	Lack of blinding<br>Statistical tests inappropriate or missing<br>Missing baseline information<br>Intervention varied or poorly described<br>No control group<br>Lack of clinical outcome measure<br>Not a fixed cohort

**Morrison,[24] 2009**	
Study title	Antipsychotic prescribing in nursing homes: an audit report
Study design	Before-after
Location	UK
Setting	Three nursing homes
Number of participants	*n*=22

© 2018 Sheehan R et al. *JAMA Network Open.*

Participant characteristics	Adults with dementia living in nursing home accommodation
Intervention	Application of a structured antipsychotic medication review (including rationale for prescription, assessment of side-effects, and consideration of dose reduction or discontinuation)
Duration of follow-up	6 months
Optimisation outcome measures and results	**Number (proportion) of participants prescribed antipsychotic drugs before and after medication review**<br>Reduced from 22 (27%) at baseline to 15 (19%) at follow-up
Author's conclusions	The introduction of a checklist and regular medication review ensures appropriate use of antipsychotic medication in this group
Source of funding	Not given
Quality assessment	Poor
Critical appraisal	Lack of blinding<br>Small sample<br>Statistical tests inappropriate or missing<br>Missing baseline information<br>Lack of control group<br>Lack of clinical outcome measure

**Napolitano et al,[25] 2012**	
Study title	Review clinic for people receiving anti-depressants
Study design	Before-after
Location	UK
Setting	Primary care
Number of participants	*n*=32
Participant characteristics	People prescribed anti-depressant medication for depression (with or without anxiety) for ≥2 years
Intervention	Face-to-face medication review by nurse prescriber to address medication and illness-related variables and included data from standardized scales (PHQ[a], GAD[b], WASAS[c], PS[d])
Duration of follow-up	Unclear
Optimisation outcome measures and results	**Number (proportion) of participants with change in anti-depressant prescription following medication review**<br>15 (47%) stopped anti-depressant medication<br>14 (44%) no change<br>3 (9%) increase dose
Author's conclusions	A nurse-led medication review clinic is effective in improving the quality of care and clinical outcomes
Source of funding	Not given
Quality assessment	Poor
Critical appraisal	Selection bias<br>Lack of blinding<br>Missing baseline information<br>Small sample<br>Statistical tests inappropriate or missing<br>Lack of control group<br>Lack of clinical outcome measure<br>Short follow-up

[a]PHQ, patient health questionnaire; [b]GAD, generalised anxiety disorder scale; [c]WASAS, work and social adjustment scale; [d]PS, phobic scale

**Prentice et al,[26] 2014**	
Study title	Reducing antipsychotic drugs in care homes
Study design	Before-after
Location	UK
Setting	463 nursing homes
Number of participants	*n*=3,165
Participant characteristics	Older adults living in nursing homes<br>41.1% had recorded dementia

© 2018 Sheehan R et al. *JAMA Network Open.*

Intervention	Pharmacist medication review based on NICE[a] guidance. Reviewed symptoms (indications), side-effects, risk:benefit ratio and discussed potential for antipsychotic reduction or discontinuation with care home staff before making recommendations for prescriber (GP or psychiatrist).
Duration of follow-up	2-4 months
Optimisation outcome measures and results	**Number (proportion) of antipsychotic dose reductions in those undergoing review**<br>653/3,165 (20.6%)<br>**Number (proportion) of antipsychotic drug discontinuations in those undergoing review**<br>548/3,165 (17.3%)
Author's conclusions	A large reduction in antipsychotic prescribing was achieved with active intervention of community pharmacists
Source of funding	Boots UK (pharmacy chain)
Quality assessment	Poor
Critical appraisal	Lack of blinding<br>Statistical tests inappropriate or missing<br>Missing baseline information<br>Intervention varied or poorly described<br>Lack of control group<br>Lack of clinical outcome measure<br>Possible conflict of interest in funding

[a]NICE, National Institute for Health and Care Excellence

**Seltzer et al,[27] 2000**	
Study title	Drug utilization review of sedative / hypnotic agents in Texas Medicaid patients
Study design	Before-after
Location	USA
Setting	Community
Number of participants	*n*=244
Participant characteristics	Age range 7-95 years (61% >60 yrs)<br>71% female
Intervention	Two stage review; automated medication review to flag potentially inappropriate sedative/hypnotic prescribing (excessive dose, extended therapy, sedative / hypnotic polypharmacy) followed by letter to prescriber (physician) prompting medication review
Duration of follow-up	12 months
Optimisation outcome measures and results	**Number (proportion) of potentially inappropriate prescriptions stopped at follow-up**<br>37/244 (15%) prescriptions for potentially inappropriate sedative/hypnotic agents stopped
Author's conclusions	Drug utilisation review can be useful in encouraging physicians to modify prescribing practices
Source of funding	Not given
Quality assessment	Fair
Critical appraisal	Selection bias<br>Lack of blinding<br>Missing baseline information<br>Intervention varied or poorly described<br>No control group<br>Lack of clinical outcome

© 2018 Sheehan R et al. *JAMA Network Open.*

eTable 3. Economic Measures and Outcomes of Medication Review

Study	Economic measure	Result	Author's conclusion
Jordan et al,[3] 2015	Direct cost of performing medication review	31.50 USD (20.50 GBP, 28.20 EUR) (2015)	The medication review was low cost and offers potential for cost savings
Johnson et al,[20] 2012	Reduction in drug prescribing costs following drug changes as a result of medication review	Reduced prescribing costs of 23,320 GBP per year (2012)	Medication review provided reductions and prescribing and possible associated costs
Napolitano et al,[25] 2012	Reduction in drug prescribing costs following drug changes as a result of medication review	Potential savings of 17,512 GBP over two years (2012)	Savings can be made with timely and appropriate medication review
Marcoux,[23] 1985	Reduction in drug prescribing costs following drug changes as a result of medication review	Estimated savings of 2,800 to 3,200 USD over a 10 month period (1985)	Medication review can result in savings
Ellenor and Frisk,[13] 1977	Reduction in drug prescribing costs following drug changes as a result of medication review	Net savings of 10,176 USD per year (1977)	A reduction in drug expenditure can be achieved with medication review

Figures given as reported by study and not adjusted for inflation.

© 2018 Sheehan R et al. *JAMA Network Open.*

eReferences

1. Ballard C, Orrell M, YongZhong S, et al. Impact of antipsychotic review and nonpharmacological intervention on antipsychotic use, neuropsychiatric symptoms, and mortality in people with dementia living in nursing homes: a factorial cluster-randomized controlled trial by the Well-Being and Health for People With Dementia (WHELD) program. *Am J Psychiatry*. 2016;173(3):252-262. Medline:26585409 doi:10.1176/appi.ajp.2015.15010130

2. Ballard C, Orrell M, Sun Y, et al. Impact of antipsychotic review and non-pharmacological intervention on health-related quality of life in people with dementia living in care homes: WHELD—a factorial cluster randomised controlled trial. *Int J Geriatr Psychiatry*. 2017;32(10):1094-1103. Medline:27640872 doi:10.1002/gps.4572

3. Jordan S, Gabe-Walters ME, Watkins A, et al. Nurse-led medicines' monitoring for patients with dementia in care homes: a pragmatic cohort stepped wedge cluster randomised trial. *PLoS One*. 2015;10(10):e0140203. Medline:26461064 doi:10.1371/journal.pone.0140203

4. Moncrieff J, Azam K, Johnson S, et al. Results of a pilot cluster randomised trial of the use of a medication review tool for people taking antipsychotic medication. *BMC Psychiatry*. 2016;16:205. Medline:27377549 doi:10.1186/s12888-016-0921-7

5. Patterson SM, Hughes CM, Crealey G, Cardwell C, Lapane KL. An evaluation of an adapted US model of pharmaceutical care to improve psychoactive prescribing for nursing home residents in Northern Ireland (Fleetwood Northern Ireland Study). *J Am Geriatr Soc*. 2010;58(1):44-53. Medline:20002510 doi:10.1111/j.1532-5415.2009.02617.x

6. Bach LL, Lazzaretto DL, Young CF, Lofholm PW. Improving nursing home compliance via revised antipsychotic use survey tool. *Consult Pharm*. 2017;32(4):228-238. Medline:28376988 doi:10.4140/TCP.n.2017.228

7. Bisconer SW, Zhang X, Sine LF. Impact of a psychotropic medication and physical restraint review process on adults with mental retardation, psychiatric diagnoses, and challenging behaviors. *J Dev Phys Disabil*. 1995;7(2):123-135. doi:10.1007/BF02684957

8. Branford D. A review of antipsychotic drugs prescribed for people with learning disabilities who live in Leicestershire. *J Intellect Disabil Res*. 1996;40(pt 4):358-368. Medline:8884591 doi:10.1111/j.1365-2788.1996.tb00641.x

9. Child A, Clarke A, Fox C, Maidment I. A pharmacy led program to review anti-psychotic prescribing for people with dementia. *BMC Psychiatry*. 2012;12:155. Medline:23006528 doi:10.1186/1471-244X-12-155

10. Craig TJ, Mehta RM. Clinician-computer interaction: automated review of psychotropic drugs. *Am J Psychiatry*. 1984;141(2):267-270. Medline:6691492 doi:10.1176/ajp.141.2.267

11. Dahl LJ, Wright R, Xiao A, Keeven A, Carr DB. Quality improvement in long term care: the Psychotropic Assessment Tool (PAT). *J Am Med Dir Assoc*. 2008;9(9):676-683. Medline:18992701 doi:10.1016/j.jamda.2008.07.002

12. Donat DC. Impact of a clinical-administrative review procedure on reducing reliance on psychotropic PRN medication. *Psychiatr Rehabil J*. 2006;29(3):215-218. Medline:16450933 doi:10.2975/29.2006.215.218

13. Ellenor GL, Frisk PA. Pharmacist impact on drug use in an institution for the mentally retarded. *Am J Hosp Pharm*. 1977;34(6):604-608. Medline:879182

© 2018 Sheehan R et al. *JAMA Network Open.*

14. Ferguson DG, Cullari S, Davidson NA, Breuning SE. Effects of data-based interdisciplinary medication reviews on the prevalence and pattern of neuroleptic drug use with institutionalized mentally retarded persons. *Educ Train Ment Retard*. 1982;17(2):103-108.

15. Gallimore CE, Sokhal D, Zeidler Schreiter E, Margolis AR. Pharmacist medication reviews to improve safety monitoring in primary care patients. *Fam Syst Health*. 2016;34(2):104-113. Medline:26986622 doi:10.1037/fsh0000185

16. Gemelli MG, Yockel K, Hohmeier KC. Evaluating the impact of pharmacists on reducing use of sedative/hypnotics for treatment of insomnia in long-term care facility residents. *Consult Pharm*. 2016;31(11):650-657. Medline:28107122 doi:10.4140/TCP.n.2016.650

17. Glaser BA, Morreau LE. Effects of interdisciplinary team review on the use of antipsychotic agents with severely and profoundly mentally retarded persons. *Am J Ment Defic*. 1986;90(4):371-379. Medline:3946474

18. Inoue F. A clinical pharmacy service to reduce psychotropic medication use in an institution for mentally handicapped persons. *Ment Retard*. 1982;20(2):70-74. Medline:7078402

19. Jauernig R, Hudson A. Evaluation of an interdisciplinary review committee managing the use of psychotropic medication with people with intellectual disabilities. *J Intellect Dev Disabil*. 1995;20(1):51-61.

20. Johnson CF, Macdonald HJ, Atkinson P, Buchanan AI, Downes N, Dougall N. Reviewing long-term antidepressants can reduce drug burden: a prospective observational cohort study. *Br J Gen Pract*. 2012;62(604):e773-e779. Medline:23211181 doi:10.3399/bjgp12X658304

21. Laska E, Siegel C, Simpson G. Automated review system for orders of psychotropic drugs. *Arch Gen Psychiatry*. 1980;37(7):824-827. Medline:7396662 doi:10.1001/archpsyc.1980.01780200102013

22. Lepler S, Hodas A, Cotter-Mack A. Implementation of an interdisciplinary psychotropic drug review process for community-based facilities. *Ment Retard*. 1993;31(5):307-315.

23. Marcoux AW. Implementation of a psychotropic drug review service in a mental retardation facility. *Hosp Pharm*. 1985;20(11):827-831. Medline:10274172

24. Morrison A. Antipsychotic prescribing in nursing homes: an audit report. *Qual Prim Care*. 2009;17(5):359-362. Medline:20003722

25. Napolitano J, Hirst C, Oldknow H. Review clinic for people receiving antidepressants. *Ment Health Pract*. 2012;16(4):10-14. doi:10.7748/mhp2012.12.16.4.10.c9465

26. Prentice A, Wright D. Reducing antipsychotic drugs in care homes. *Nurs Times*. 2014;110(23):12-15. Medline:25016733

27. Seltzer JK, Kurt TL, Knodel LC, Dean B, Burch C; Texas Medicaid Vendor Drug Program Drug Utilization Review Board. Drug utilization review of sedative/hypnotic agents in Texas Medicaid patients. *J Am Pharm Assoc (Wash)*. 2000;40(4):495-499. Medline:10932458

© 2018 Sheehan R et al. *JAMA Network Open.*
